# Sensory-Driven Formulation Optimization, Physicochemical Properties, Antioxidant Activity, and Structural Characterization of Erythritol-Based Green Tea Cookies

**DOI:** 10.3390/foods15213776

**Published:** 2026-10-22

**Authors:** Luna Zhu, Jiale Huo, Feng Hu, Fuqiang Qi, Yao Xiao, Manzhao Yan, Qingshan Xu, Youben Yu, Jie Zhou

**Affiliations:** 1 College of Horticulture, Northwest A&F University, Taicheng Road, Xianyang 712100, China; 2 Shaanxi Horticultural Technology Station, Xi’an 710018, China; 3 Xixiang County Tea Industry Development Center, Hanzhong 723500, China

**Keywords:** green tea powder, sugar substitute, response surface methodology, texture, microstructure, functional food

## Abstract

Although green tea is a well-known source of polyphenols with pronounced antioxidant properties, few studies have explored its use in erythritol-based baked products. In this study, an erythritol-based green tea cookie (GTC) was developed using green tea powder (GTP), butter, and erythritol. The formula was then optimized by single-factor experiments and a Box–Behnken response surface design. The optimal formulation contained 7.88 g GTP, 72.13 g butter, and 39.63 g erythritol, with a predicted sensory score of 80.35, while the validated sensory score was 81.07 ± 1.87 as evaluated by 12 trained panelists. The optimized cookies exhibited a 4.5-fold increase in total phenolic content compared with the control butter cookies (BC) and significantly enhanced antioxidant activity, with a ferric reducing antioxidant power (FRAP) value of 96.06 mg AAE/g dry weight (DW). GTP also modified the color and textural attributes of the cookies. Scanning electron microscopy (SEM) revealed a denser and more homogeneous microstructure than that of the control butter cookies (BC), with the pore area fraction reduced from 17.8% to 8.0%. Fourier transform infrared (FTIR) analysis indicated a redistribution of protein secondary structural elements, suggesting possible structural rearrangement. Overall, these findings demonstrate that GTP enhances the antioxidant capacity and modifies the quality attributes of erythritol-based cookies, providing a potential approach for developing functional erythritol-based products.

## 1. Introduction

Owing to their crispy texture and characteristic appearance, cookies have become one of the most widely consumed bakery snacks worldwide [1]. As public awareness of health and wellness grows, traditional baked goods—typically high in sugar, fat, and energy—are being reformulated into functional, reduced-sugar products that preserve desirable sensory and nutritional value [2]. Against this backdrop, the development of sugar-reformulated functional cookies fortified with natural bioactive compounds has attracted increasing research interest in the healthy food industry [3,4,5,6,7]. In recent years, many researchers have incorporated various bioactive ingredients such as tea powder, fruit and vegetable powders, and plant extracts into cookie formulations to improve their functional properties [3,8,9,10,11].

Green tea powder (GTP) is a concentrated source of tea polyphenols, L-theanine, purine alkaloids, and dietary fibers, exhibiting excellent health benefits and great application potential in baked goods such as bread and cookies, as well as noodle products [5,12]. Previous studies have shown that appropriate supplementation of GTP can effectively increase the total phenolic content and enhance the antioxidant activity of baked goods [13,14]. Therefore, properly controlling the GTP addition amount and optimizing processing parameters are crucial for achieving synergistic improvements in the quality and functional properties of baked goods. It is worth noting that the development of low-sugar baked goods requires not only the incorporation of functional ingredients but also the reformulation of the sweetening system, which is a key step for achieving the low-sugar goal [15]. Erythritol, a natural polyol sweetener, delivers a clean sweet taste with virtually no caloric contribution and does not raise blood glucose levels [16,17,18]. However, the partial or total replacement of sucrose with erythritol may alter the rheological behavior, texture properties, and sensory characteristics of the dough, highlighting the necessity of systematic formulation optimization for erythritol-based cookie products [19]. For baked goods, sensory quality is a primary determinant of product acceptance, with flavor, texture, and appearance serving as the key evaluation criteria [5,13,20]. While functional ingredients give products additional nutritional value, inferior sensory quality will greatly limit their acceptability [5]. Therefore, sensory performance remains an indispensable evaluation criterion for the formulation optimization of functional cookies [20,21].

Although GTP-fortified cookies and erythritol-based bakery products have been investigated independently [13,22], three gaps remain in this field. First, the combination of GTP and erythritol in a single cookie system has rarely been explored, and most existing studies focused on only one of these strategies. Second, formulation optimization has generally been driven by physicochemical indicators, with sensory acceptance—the decisive criterion for product acceptance—often assessed separately or as a secondary response rather than as the primary optimization target [3,11,23,24]. Third, the mechanistic basis linking formulation changes to textural and microstructural variations has seldom been examined in a multi-scale manner. To the best of our knowledge, studies that combine GTP–erythritol co-formulation, sensory-driven response surface methodology (RSM) optimization, and integrated physicochemical, SEM, and FTIR characterization in a single cookie system remain scarce. This study was therefore designed to fill these gaps by developing an erythritol-based green tea cookie (GTC) through sensory-driven RSM optimization and by systematically characterizing the optimized product from the physicochemical to the structural level.

To fill the above research gaps, this study developed an erythritol-based green tea cookie (GTC) with erythritol as the sucrose substitute and GTP as the core functional ingredient. With sensory quality set as the primary optimization target, the formulation was refined by single-factor experiments and subsequently by RSM [24]. Subsequently, the optimized GTC was systematically compared with the control butter cookie (BC) in terms of nutritional components, color, texture, tea polyphenol content, and antioxidant activity. Furthermore, SEM and FTIR were used to probe the microstructural origins of the quality differences between the two cookies. Overall, this work is expected to offer practical and scientific reference for developing GTP-fortified functional baked goods sweetened with erythritol.

## 2. Materials and Methods

### 2.1. Materials

Ultra-fine green tea powder (GTP, approx. 1200 mesh) was purchased from Jiangsu Xinpin Tea Co., Ltd. (Changzhou, Jiangsu, China). Low-gluten wheat flour (protein: 8.9 g/100 g, fat: 1.2 g/100 g, carbohydrate: 75.4 g/100 g, as declared on the product label), erythritol (purity ≥ 99%), milk powder, and butter were purchased from a baking supplier (ZhanYi, Shanghai, China), and fresh chicken eggs were obtained from a local market in Yangling, Shaanxi Province, China.

### 2.2. Cookie Preparation

#### 2.2.1. Basic Formulation of Cookies

The basic cookie formulation of green tea cookie (GTC) consisted of 135 g low-gluten wheat flour, 5 g GTP, 30 g erythritol, 30 g whole egg liquid, 75 g butter, 1 g salt, and 3 g milk powder. In all formulations, GTP was incorporated as an equal-weight partial replacement of low-gluten wheat flour, i.e., the total weight of the flour–GTP mixture was kept constant at 140 g.

#### 2.2.2. Preparation Process of Green Tea Cookie

**Figure 1:**
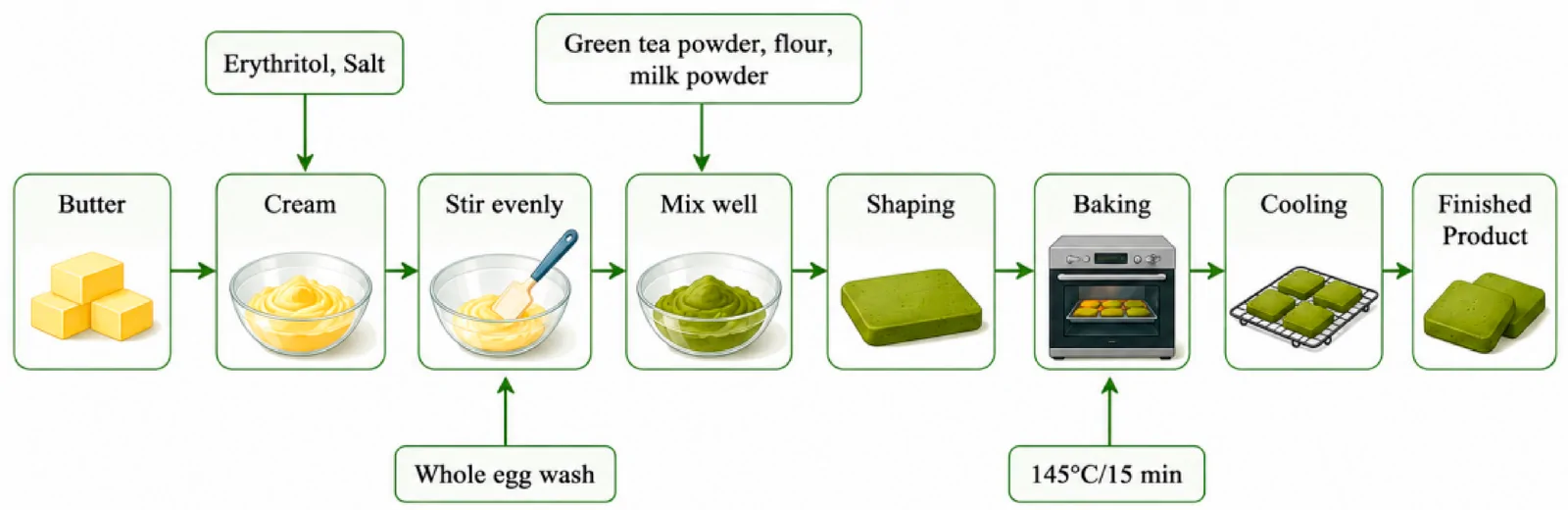
Process flow for the preparation of green tea cookies.

The main steps of GTC preparation are shown in Figure 1. In brief, butter was first softened at room temperature and creamed together with erythritol and salt under continuous stirring. Whole egg liquid was then added slowly and blended until homogeneous. Subsequently, GTP, low-gluten wheat flour, and milk powder were added and kneaded using a dough mixer (Royalstar RM1200Z, Hefei, China) at speed level 2 for 2–3 min to form a dough. The dough was shaped and sliced into uniform pieces (4 cm × 4 cm × 2 mm) and baked in a preheated oven at 145 °C for 15 min. Following baking, the products were cooled to room temperature, stored in airtight packaging, and held for further testing.

The cookies used in the experiments of Section 3.3, Section 3.4, Section 3.5, Section 3.6 and Section 3.7 were prepared using the response-surface-optimized GTC formulation (7.9 g GTP, 39.6 g erythritol, 72.1 g butter, and 132.1 g low-gluten wheat flour). For the control butter cookies (BC), the same procedure was followed, except that the 7.9 g of GTP was substituted with an equivalent amount (7.9 g) of low-gluten wheat flour, so that BC contained 140.0 g of wheat flour in total.

#### 2.2.3. Single-Factor Experiment

Four key variables were evaluated individually in single-factor experiments, including GTP addition amount (2.5, 5.0, 7.5, 10.0, 12.5, and 15.0 g), erythritol addition amount (10, 20, 30, 40, 50, and 60 g), butter addition amount (45, 55, 65, 75, 85, and 95 g), and baking conditions (temperature and time) on the sensory quality of GTC. The investigated factors and their corresponding levels are listed in Table 1. In each single-factor experiment, only one factor was varied across its six levels, while all other parameters were held constant at the basic formulation levels established in Section 2.2.1 (5 g GTP, 30 g erythritol, 75 g butter, 145 °C, 15 min). For the baking condition factor, six paired temperature–time combinations (125 °C/22 min, 135 °C/18 min, 145 °C/15 min, 155 °C/13 min, 165 °C/11 min, and 175 °C/8 min) were examined to evaluate the combined effects of baking temperature and time, and their individual effects were not evaluated separately. The sensory scores were adopted as the primary criterion for evaluating product quality. For each factor, the level achieving the highest sensory score was selected as the optimal level, which was then adopted as the fixed condition for the subsequent experiment. Sensory evaluation was conducted once by 12 trained panelists, and the sensory scores were expressed as the mean ± SD (*n* = 12), reflecting inter-panelist variation.

#### 2.2.4. Response Surface Experiment

Following the single-factor results, a Box–Behnken response surface design with three factors and three levels was adopted, comprising 17 runs in total, five of which were center-point replicates. The runs were performed according to the standard order of the design, and the samples were presented to the panelists in a randomized order for sensory evaluation. The contents of GTP, erythritol, and butter served as the independent variables, with the comprehensive sensory score taken as the response. The corresponding factor levels are given in Table 1.

### 2.3. Sensory Evaluation

Sensory evaluation was performed by 12 trained panelists (half male and half female, aged 20–25 years) majoring in tea science, rather than untrained consumers [13]. Each panelist was briefed on the aims and protocol of the study and signed an informed consent form before participating. The cookies assessed here included wheat flour, egg, butter, and milk powder, and panelists who reported allergies to any of these ingredients were duly cautioned. Participation was voluntary, with the option to withdraw at any time, and no adverse events occurred during the study. Before the formal session, all panelists took part in a training session covering the scoring criteria in Table S1, to ensure consistent interpretation of the five attributes. All evaluations were carried out in separate booths illuminated with standard white light at 25 °C. Each sample was assigned a random three-digit code and served in randomized order, with the panelists unaware of the corresponding formulations. Distilled water was supplied between samples for mouth rinsing. For the single-factor experiments, each panelist evaluated six samples (two cookies of each formulation level). For the response surface experiments, the 17 formulations were evaluated in three rounds to minimize sensory fatigue. A weighted scoring system was used for five attributes—shape and appearance, color, aroma, taste, and texture—each scored on a 20-point scale according to the criteria in Table S1, and the comprehensive score was calculated as the weighted sum of the attribute scores multiplied by 5, giving a maximum of 100 points.

$$\begin{array}{l} Comprehensive\;sensory\;score\\ \;=5\times [0.20\times S(shape\;and\;appearance)+0.15\times S(color)\\ \;+0.15\times S(aroma)+0.30\times S(taste)+0.20\times S(texture)] \end{array}$$

where S(attribute) denotes the score of each attribute. The sensory ballot used in this study is provided in Table S2. The mean of the scores from all 12 panelists was used as the response value in the single-factor and response surface analyses. To validate the predicted optimum, the optimized formulation was additionally evaluated in three independent sessions, and the mean score was used as the validated result.

### 2.4. Determination of Basic Nutritional Components

The basic nutritional components of the cookies were analyzed according to national standard procedures. Moisture was quantified by the direct drying method (GB 5009.3-2016) [25]. Crude protein was measured with a fully automated Kjeldahl analyzer following the Kjeldahl method (GB 5009.5-2025) [26], using a nitrogen-to-protein conversion factor of 6.25. Crude fat was determined by Soxhlet extraction (GB 5009.6-2025) [27], and ash by incineration in a muffle furnace at 550 °C (GB 5009.4-2016) [28]. Total carbohydrates were calculated using the difference method (Equation (1)), and total energy was calculated based on the Atwater coefficient system (Equation (2)). The erythritol content was calculated from the amount added in the formulation and the moisture loss during baking. No individual carbohydrate fractions (e.g., starch, sugars, and dietary fiber) were separately determined.

(1) Total carbohydrate(g/100 g)=100−(moisture+protein+fat+ash)

(2) $$\begin{array}{l} Energy(kcal/100\;g)\\ \;=[total\;carbohydrate(g/100\;g)\\ \;-erythritol(g/100\;g)]\times 4(kcal/g)\\ \;+[crude\;protein(g/100\;g)\times 4(kcal/g)]\\ \;+[crude\;fat(g/100\;g)\times 9(kcal/g)] \end{array}$$

### 2.5. Determination of Physical Properties

Color measurements were performed with a colorimeter (Ci7600, X-Rite, Grand Rapids, MI, USA). Following calibration against a standard white plate, the L* (lightness), a* (redness/greenness), and b* (yellowness/blueness) values of the samples were recorded. For color measurement, three cookies from the same batch were used, with one measurement taken on each cookie, and the average value was calculated.

Texture was evaluated with a texture analyzer (TA.XT Plus, Stable Micro Systems, Godalming, UK) fitted with a three-point bending rig (HDP/3PB) at a support span of 30 mm. Hardness, flexibility, and breaking work were derived from the force–time curve of the three-point bending test, as the force at the maximum positive peak, the distance at that peak, and the area under the curve up to that peak, respectively. Hardness was expressed in gram-force (g), flexibility in millimeters (mm), and breaking work in g·s, as directly obtained from the instrument. The conversion factors are 1 g = 0.00980665 N and 1 g·s = 9.80665 × 10^−3^ N·s. Test conditions: pre-test speed 2.5 mm/s, during-test speed 2.0 mm/s, post-test speed 10.0 mm/s, deformation distance 15.0 mm, contact force 0.2 N, data acquisition rate 500 pps. Six cookies from the same batch were analyzed.

### 2.6. Total Phenolic Content and In Vitro Antioxidant Activity Analysis

Total phenolic content (TPC) was assayed by the Folin–Ciocalteu procedure with minor modifications, following GB/T 8313-2018 [29]. Briefly, 1.0 mL of the extract was combined with 5.0 mL of 10% Folin–Ciocalteu reagent, vortexed, and left for 3–8 min; 4.0 mL of 7.5% sodium carbonate solution was then added, and the mixture was brought to 25 mL with water and kept for 60 min at room temperature in the dark. Absorbance was read at 765 nm against a gallic acid standard curve. TPC was calculated from the linear regression of that curve and expressed as g gallic acid equivalents (GAE)/100 g DW.

For extract preparation, 0.5 g of freeze-dried cookie powder was weighed out and mixed with 5 mL of 80% ethanol. The mixture was extracted in a water bath at 65 °C for 15 min, followed by centrifugation at 4000 rpm for 10 min. This extraction step was repeated once, and the combined supernatants were made up to a final volume of 10 mL. The resulting extract was stored at 4 °C away from light until use.

DPPH and ABTS^+^ radical scavenging capacities and total antioxidant capacity (FRAP) were determined according to the methods of Brand-Williams et al., Re et al. [30,31], and Benzie and Strain [32], respectively, with slight modifications. The procedures followed the cited methods with the following conditions: DPPH (100 μL extract + 100 μL 0.1 mmol/L DPPH solution, 25 °C in the dark for 30 min, 517 nm), ABTS^+^ (working solution prepared by mixing equal volumes of 7.4 mmol/L ABTS solution and 2.6 mmol/L potassium persulfate, kept in the dark for 14 h and diluted with anhydrous ethanol to an absorbance of 0.70 ± 0.02 at 734 nm, 20 μL extract + 180 μL working solution, 37 °C in the dark for 6–10 min, 734 nm), with the scavenging rate calculated according to Equation (3). For FRAP, the reagent was freshly prepared as a 10:1:1 (*v*/*v*/*v*) mixture of acetate buffer (300 mmol/L, pH 3.6), TPTZ (10 mmol/L in 40 mmol/L HCl), and FeCl_3_·6H_2_O (20 mmol/L); 10 μL of extract and 190 μL of reagent were mixed, incubated for 15 min in the dark at room temperature, and the absorbance was measured at 593 nm. Results were expressed as ascorbic acid equivalents (mg AAE/g DW) (Equation (4)).

(3) $$Scavenging\;rate(\%)=[1-(A_{1}-A_{2})/A_{0}]\times 100$$

(4) AAE(mgAAE/gDW)=C×V/m

where A_0_, A_1_, and A_2_ are the absorbances of the control (extract replaced by anhydrous ethanol), the sample (extract + DPPH/ABTS^+^ working solution), and the sample blank (DPPH/ABTS^+^ solution replaced by anhydrous ethanol), respectively. C is the ascorbic acid equivalent concentration (mg/mL) read from the linear regression of the ascorbic acid standard curve (A = aC + b); V is the total extract volume; and m is the mass of the freeze-dried cookie powder.

### 2.7. Scanning Electron Microscopy (SEM) Analysis

The cookie samples were cut into approximately 5 mm × 5 mm × 2 mm pieces, placed in centrifuge tubes, defatted by soaking in petroleum ether (boiling range 30–60 °C) for 12 h with the solvent replaced 2–3 times, and freeze-dried under vacuum. A naturally fractured cross-section was mounted on the SEM stage with conductive adhesive and sputter-coated with gold (GV1000, Gewei, Beijing, China; 10 mA, 90 s) [33]. Samples were examined by field-emission scanning electron microscopy (SU5000, Hitachi, Tokyo, Japan) at 3.0 kV, with images captured at 250× for porosity quantification and at 1000× for microstructure observation. Porosity was quantified from SEM images captured at a magnification of 250×. For each treatment, three images were acquired from different regions of a single cookie specimen to assess structural homogeneity. In ImageJ 1.53c (National Institutes of Health, Bethesda, MD, USA), images were converted to 8-bit grayscale, and the threshold was first set automatically using the Otsu method [34] and then manually refined to match the pore boundaries visible in the original image. Porosity was calculated as the ratio of pore area to total image area (%) [35].

### 2.8. Fourier Transform Infrared (FTIR) Spectroscopy Analysis

FTIR spectra were acquired in attenuated total reflection (ATR) mode with a Bruker INVENIO R spectrometer (Bruker, Ettlingen, Germany) [36]. Freeze-dried cookie powder was pressed against the diamond crystal. Spectra were collected at 400–4000 cm^−1^ with air as background (re-acquired before each sample) and converted from transmittance to absorbance using OMNIC 9.8 (Thermo Fisher Scientific, Waltham, MA, USA). The amide I region (1600–1700 cm^−1^) was analyzed in PeakFit 4.12 (Systat Software, San Jose, CA, USA), where baseline correction was performed automatically, followed by peak deconvolution, with peak positions, widths, and amplitudes freely varied during fitting. The deconvoluted components were identified as β-sheet (1610–1640 cm^−1^), random coil (1640–1650 cm^−1^), α-helix (1650–1660 cm^−1^), and β-turn (1660–1670 cm^−1^), and the proportion of each was obtained from the integrated peak areas (%) [37]. One representative spectrum was analyzed per treatment. Goodness of fit was *R*^2^ > 0.90 for all deconvolutions.

### 2.9. Statistical Analysis

Each physicochemical measurement was carried out three times on individual cookie subsamples from the same batch, and the data are presented as the mean ± standard deviation (mean ± SD). Texture measurements were performed in six replicates (*n* = 6 per group). Sensory evaluation was conducted once by 12 trained panelists, and the sensory scores were expressed as the mean ± SD (*n* = 12). The replicate measurements were used to estimate within-formulation variability, and all statistical comparisons reflect differences within the studied batch rather than reproducibility across independently prepared batches. Before parametric analysis, the normality (Shapiro–Wilk test and Q–Q plots) and homogeneity of variance (Levene’s test) of the data were checked using SPSS 26.0. For two-group comparisons (e.g., BC vs. GTC), differences were analyzed using an independent-samples *t*-test. Data from single-factor experiments with more than two levels were analyzed by one-way ANOVA followed by Duncan’s multiple range test when the underlying assumptions held. When normality was violated, the Mann–Whitney U test was used instead. Differences were considered significant at *p* < 0.05, and Origin 2024 was employed for data processing and figure generation.

## 3. Results and Discussion

### 3.1. Effects of Single-Factor Experiments on the Sensory Quality of GTC

**Figure 2:**
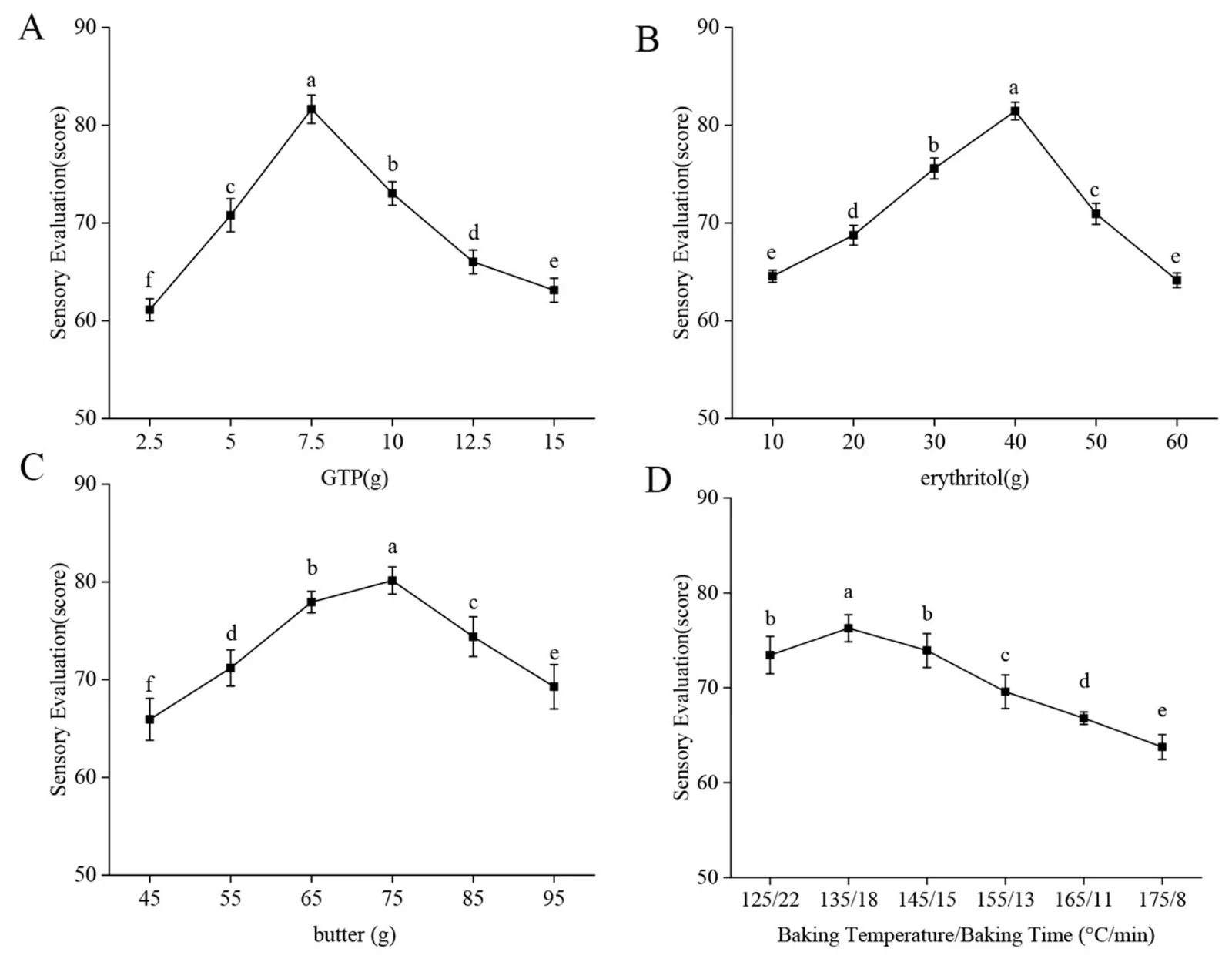
Effects of single-factor experiments on the sensory quality of green tea cookies (GTC). (**A**) Addition amount of green tea powder; (**B**) Addition amount of erythritol; (**C**) Addition amount of butter; (**D**) Baking temperature and time. Different letters indicate significant differences (*p* < 0.05).

In GTC formulations, GTP not only imparts a unique tea aroma and flavor to the product but also markedly influences the cookies’ color, flavor, and texture due to its rich content of active components such as tea polyphenols and amino acids [13]. Figure 2A shows that, as the GTP content increased, the sensory scores of the GTC first rose and then declined, reaching a peak at 7.5 g. This trend may be explained by the sensory properties associated with GTP addition: an appropriate amount of GTP imparts a refreshing tea aroma and enriches the flavor profile of the cookies. However, excessive GTP increases the tea polyphenol content, which may enhance bitter and astringent tastes and may increase cookie hardness, thereby reducing sensory acceptability [38]. Therefore, GTP levels of 5.0 g, 7.5 g, and 10.0 g were selected for subsequent response surface experiments.

As shown in Figure 2B, the sensory scores first rose and then declined with increasing erythritol content, peaking at 40 g. This may be attributed to its role in balancing sweetness and harmonizing flavors in cookie formulations [22]. At low erythritol levels, the product lacked sufficient sweetness, and the bitterness of the GTP was prominent. Simultaneously, the cookie texture was hard and had a noticeable powdery mouthfeel. At excessively high levels, the sweetness was too strong, which may mask the distinctive aroma of green tea and reduce the flavor harmony of the product. Therefore, erythritol levels of 30 g, 40 g, and 50 g were selected for subsequent response surface experiments.

Butter, as a key fat component in the cookie formulation, improves the product’s texture and imparts a crumbly mouthfeel and creamy aroma to the cookies [39]. As shown in Figure 2C, the sensory scores rose progressively with butter content, peaking at 75 g before declining. This may be related to the role of butter in dough processing: an appropriate amount of butter enhances dough processability, resulting in a smooth cookie surface, uniform texture, and crisp mouthfeel [40]. However, excessive butter increases the fat content in the dough, which may lead to an overly loose and brittle structure after baking, and the strong buttery aroma may mask the tea aroma, thereby reducing sensory quality [41]. Therefore, butter levels of 65 g, 75 g, and 85 g were selected for subsequent response surface experiments.

Baking temperature and baking time are important process factors that influence the development of cookie color, flavor release, and texture [42]. Figure 2D illustrates that the sensory scores first rose and then fell as baking conditions varied. However, the differences among treatments were relatively small, and the trend was not pronounced. It should be noted that the baking conditions were examined as paired temperature–time combinations, in which temperature and time were varied simultaneously. Consequently, their individual contributions cannot be distinguished, and the optimal conditions should be interpreted as a paired regime.

These results indicate that the baking regime affected sensory quality, but its influence was relatively limited. Accordingly, the amounts of GTP, erythritol, and butter were selected as the independent variables for the subsequent response surface optimization.

### 3.2. Response Surface Experiment and Results

Based on the results of the single-factor experiments (Figure 2), a three-factor, three-level Box–Behnken design was established (Table 2).

Using Design-Expert 13 software, a multiple regression analysis was performed on the experimental data to establish the following regression model between sensory scores and the various factors:

$$Y\;=\;80.12\;+\;1.99A-1.20B-2.24C\;+\;0.25\mathrm{AB}-0.57\mathrm{AC}-2.19\mathrm{BC}-7.12A^{2}-7.29B^{2}-3.91C^{2}$$

where Y is the comprehensive sensory score, and A, B, and C are the coded values of GTP addition, erythritol addition, and butter addition, respectively (−1, 0, +1).

The adequacy and predictive power of the response surface model were assessed by analysis of variance (Table 3). The regression model proved highly significant (*p* < 0.0001), whereas the lack of fit was not significant (*p* = 0.80), indicating that the model adequately captures the relationship between the factors and the sensory scores. The *R*^2^ was 0.98, with adjusted and predicted *R*^2^ values of 0.96 and 0.92, respectively, confirming the strong fit and predictive capacity of the model. The model’s adequacy was further confirmed by a coefficient of variation (CV) of 1.85% and an adequate precision of 17.61, together with the residual diagnostics, namely the normal probability plot of residuals and the plot of residuals versus predicted values (Table S3; Figure S1).

**Figure 3:**
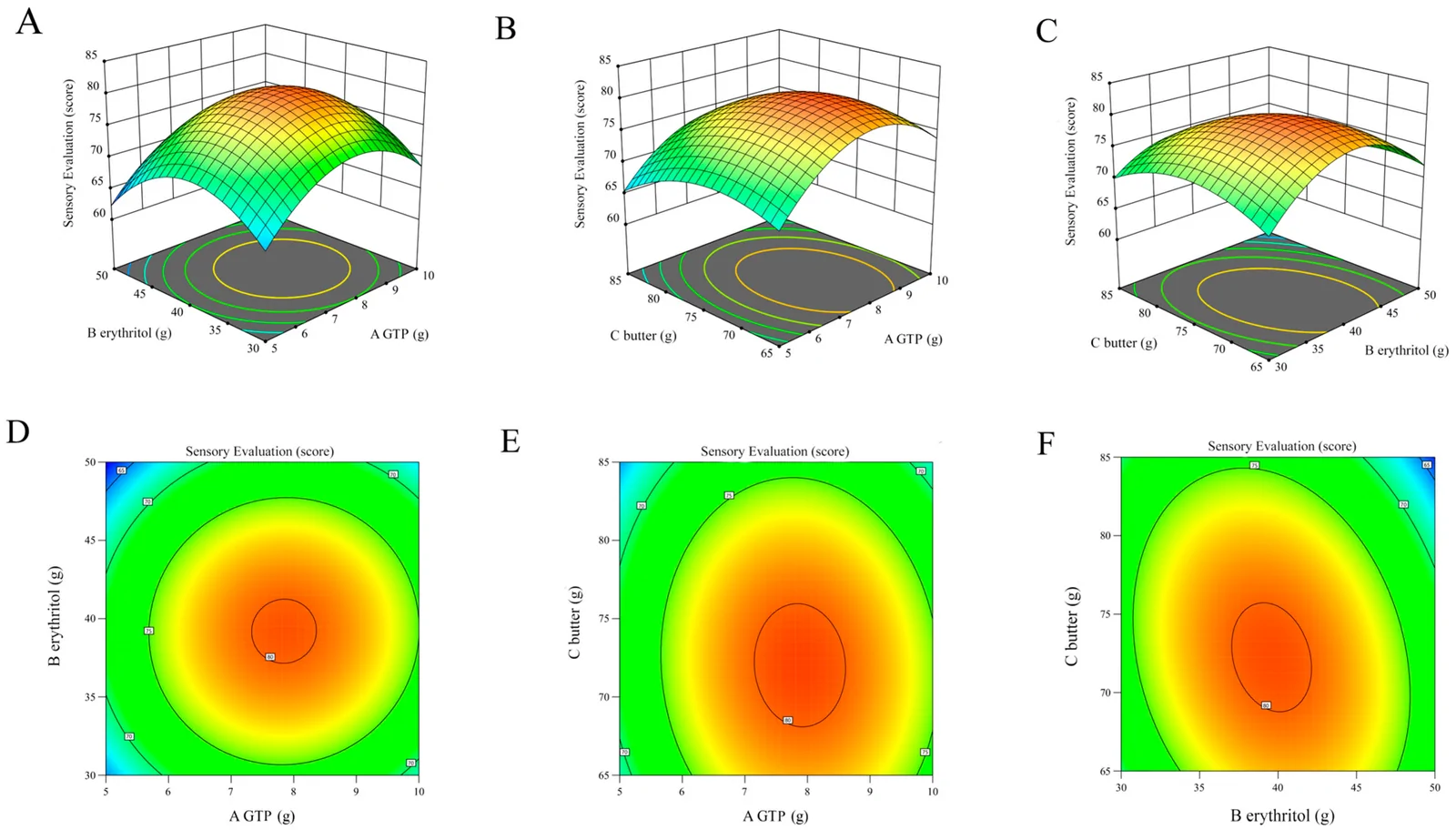
Surface and contour plots showing the interaction effects of GTP, erythritol, and butter contents on the sensory score. (**A**,**D**) 3D response surface plot and contour plot of erythritol and GTP; (**B**,**E**) 3D response surface plot and contour plot of butter and GTP; (**C**,**F**) 3D response surface plot and contour plot of erythritol and butter. The color gradient represents the magnitude of the response value, with blue indicating lower values and red indicating higher values.

According to Table 3, the sensory scores were strongly influenced by the GTP content (A), the butter content (C), and the quadratic terms A^2^, B^2^, and C^2^ (*p* < 0.01), whereas the erythritol content (B) and the interaction term BC exerted significant effects (*p* < 0.05). The surface and contour plots of the interaction effects on the sensory score are shown in Figure 3. The remaining terms had no significant effects (*p* > 0.05). Based on the *F*-values for each factor, the order of influence on sensory scores was as follows: butter content (C) > GTP content (A) > erythritol content (B). The results suggest that, within the range examined, butter content had the most significant effect on sensory scores.

The regression model was numerically optimized using Design-Expert 13 software to maximize sensory scores. The optimal formulation parameters were obtained as follows: 7.88 g of GTP, 39.63 g of erythritol, and 72.13 g of butter, with the amounts of other ingredients remaining constant. Under these conditions, the model predicted a sensory score of 80.35 points.

To verify the reliability of the model’s predictions, three independently prepared batches were produced under the optimal process conditions. Each batch was baked and sensory-evaluated in a separate session at one-week intervals to avoid sensory fatigue. The results showed that the actual sensory score was 81.07 ± 1.87 points, which was close to the model’s predicted value of 80.35 points, with a relative error of 0.90%. These results confirm that the established response surface model has good predictive capability and reliability.

### 3.3. Basic Nutritional Components

Nutrient composition is the foundation for determining the nutritional value of functional baked goods [20]. Table 4 summarizes the basic composition of BC and GTC. Relative to BC, GTC exhibited significantly higher moisture and ash contents (*p* < 0.05) and a slightly higher crude protein content, along with a significantly lower carbohydrate content (*p* < 0.05), while crude fat and calculated energy showed no marked differences. Because this substitution changes several constituents at once, the observed compositional differences cannot be attributed to any single component and should be regarded as the overall consequence of replacing part of the wheat flour with GTP.

The moisture content of GTC was considerably higher than that of BC (*p* < 0.05), which may be related to the higher dietary fiber and tea polyphenol content in GTP. These components contain numerous hydrophilic groups that can enhance water binding through hydrogen bonds, which may contribute to enhanced water retention within the cookie matrix [43]. Ash content reflects the total mineral content in food [44]. GTC showed a significantly higher ash content (*p* < 0.05), probably because tea leaves are richer in minerals such as potassium, magnesium, and calcium than wheat flour [45].

No significant difference in crude fat content was observed between BC and GTC (*p* > 0.05). Although GTP contains small amounts of lipids and other lipid-soluble constituents, it had only a limited effect on the overall crude fat content of the cookies. This result indicates that replacing a small proportion of wheat flour with GTP did not substantially alter the lipid composition of the cookie system. Previous studies have reported that tea leaves contain considerable amounts of plant proteins and free amino acids, which can contribute to the protein content of bakery products [46]. The crude protein content of GTC was slightly higher than that of BC, although the difference did not reach statistical significance, suggesting that green tea powder provided an additional source of protein for the cookies. In practical terms, this increase is modest and, at typical serving sizes, its impact on daily protein intake is limited.

Moreover, these components may further influence the dough network structure and ultimately affect the textural characteristics of the final product. Conversely, the carbohydrate content of GTC was significantly lower than that of BC (*p* < 0.05). This difference is consistent with the formulation change, in which part of the starch-rich wheat flour was replaced by GTP, a source of non-starch polysaccharides, dietary fiber, and other functional components. It should be noted that the carbohydrate content was calculated by the difference method and thus reflects the sum of all carbohydrate fractions.

The calculated energy values of BC and GTC were comparable, with no substantial difference observed between the two formulations. This is mainly because GTP was incorporated at a relatively low level, while butter, the major contributor to the caloric value of cookies, remained unchanged. Overall, GTP incorporation modified the nutritional composition of erythritol-based cookies by increasing ash content, slightly increasing protein content, and reducing the relative carbohydrate proportion. These changes may contribute to the nutritional quality of the cookies, although further studies are needed to evaluate their potential nutritional and functional benefits.

### 3.4. Texture and Color Difference

As shown in Table 5, the addition of GTP markedly affected the color and texture characteristics of the cookies. Compared with BC, GTC exhibited a significantly lower L* value (*p* < 0.01), indicating a darker appearance. This was mainly attributed to the introduction of chlorophyll and polyphenols from the GTP. Simultaneously, the oxidation of some tea polyphenols and non-enzymatic browning reactions during baking further promoted these color changes. The a* value shifted from positive to negative, reflecting the enhanced greenness of the cookies, which is closely related to the contribution of the natural green pigments in GTP. The b* value increased significantly (*p* < 0.01), suggesting an increased yellow component and resulting in a characteristic green-yellow appearance.

In terms of texture, the hardness of the GTC was markedly higher than that of the BC (*p* < 0.01), suggesting that the addition of GTP promoted the formation of a more compact internal structure. The flexibility of the GTC increased significantly (*p* < 0.01), indicating a marked increase in toughness and a decrease in crispness. In addition, the breaking work of the GTC was significantly higher than that of the BC (*p* < 0.01), further supporting that GTP enhanced the toughness of the cookies.

Overall, GTP significantly altered the color and textural characteristics of erythritol-based cookies. These changes suggested that GTP incorporation may have altered the internal organization of the cookie matrix, which was further investigated by SEM analysis.

### 3.5. Total Phenolic Content and Antioxidant Capacity

**Figure 4:**
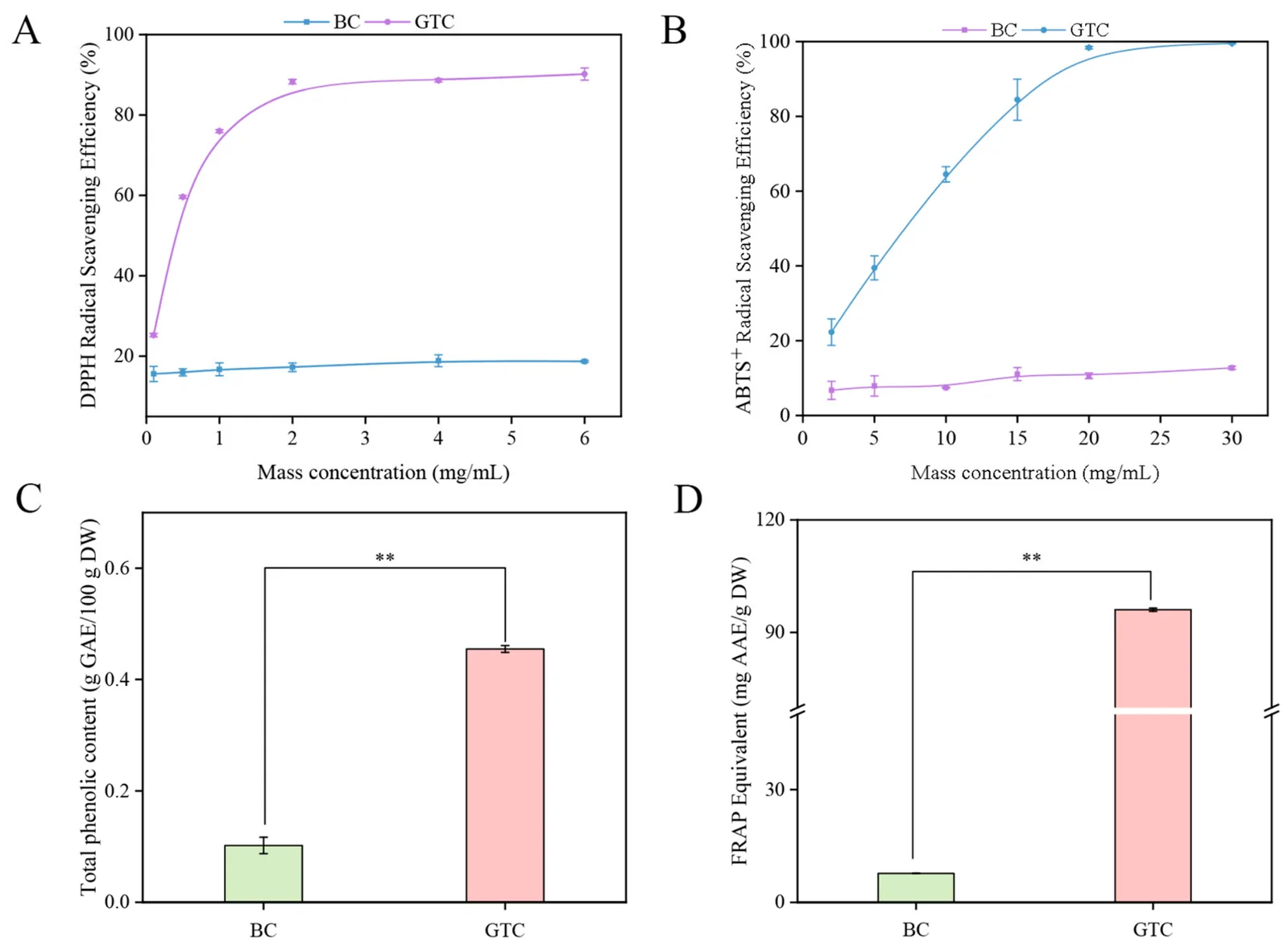
Comparison of in vitro antioxidant activity and total phenolic content between butter cookies (BC) and green tea cookies (GTC). (**A**) DPPH radical scavenging activity; (**B**) ABTS^+^ radical scavenging activity; (**C**) Total phenolic content; (**D**) Ferric reducing antioxidant power (FRAP). ** indicates significance at *p* < 0.01.

There were significant differences between GTC and BC in terms of active ingredient content and in vitro antioxidant capacity (Figure 4). The scavenging activity of GTC toward both DPPH and ABTS^+^ radicals rose in a concentration-dependent manner as the sample mass concentration increased, whereas BC remained essentially unchanged.

At equal mass concentrations, GTC showed a significantly higher DPPH radical scavenging rate than BC (Figure 4A). When the sample mass concentration reached 4 mg/mL, the DPPH scavenging rate of GTC approached 90% and tended to stabilize. GTC also exhibited strong scavenging activity toward ABTS^+^ radicals, with a continuous upward trend as the mass concentration increased (Figure 4B). By contrast, the scavenging curves of BC for both DPPH and ABTS^+^ remained relatively flat. Taken together, these findings show that the addition of GTP markedly improved the radical-scavenging capacity of the cookies.

To further characterize the antioxidant properties of the cookies, TPC and ferric reducing antioxidant power (FRAP) were measured. GTC showed significantly higher total phenolic content and ferric reducing antioxidant power (FRAP) than BC (*p* < 0.01). Specifically, the TPC in GTC reached 0.45 g GAE/100 g DW, approximately 4.5 times that of BC (Figure 4C). The FRAP value was as high as 96.06 mg AAE/g DW, whereas that of BC was only 7.71 mg AAE/g DW (Figure 4D). A possible explanation is that the active components in GTP, such as total phenolics, remained detectable after baking, providing the key molecular basis for the enhanced in vitro antioxidant capacity of the cookies [47].

Previous studies have shown that total phenolics, particularly catechin compounds, contain multiple phenolic hydroxyl groups that can scavenge free radicals through hydrogen or electron transfer mechanisms, while also inhibiting oxidative chain reactions by reducing Fe^3+^. Accordingly, the elevated TPC was accompanied by improved in vitro antioxidant capacity determined via the FRAP, DPPH, and ABTS assays, although this association should not be interpreted as causal [48,49,50]. Overall, the enhanced in vitro antioxidant activity of GTC was closely associated with its higher tea polyphenol content, highlighting the potential of GTP as a natural source of antioxidant compounds for erythritol-based products. It should also be noted that these assays evaluate in vitro antioxidant capacity only and do not directly demonstrate antioxidant effects following digestion, absorption, or in vivo metabolism.

### 3.6. Microstructure Analysis of Cookies

**Figure 5:**
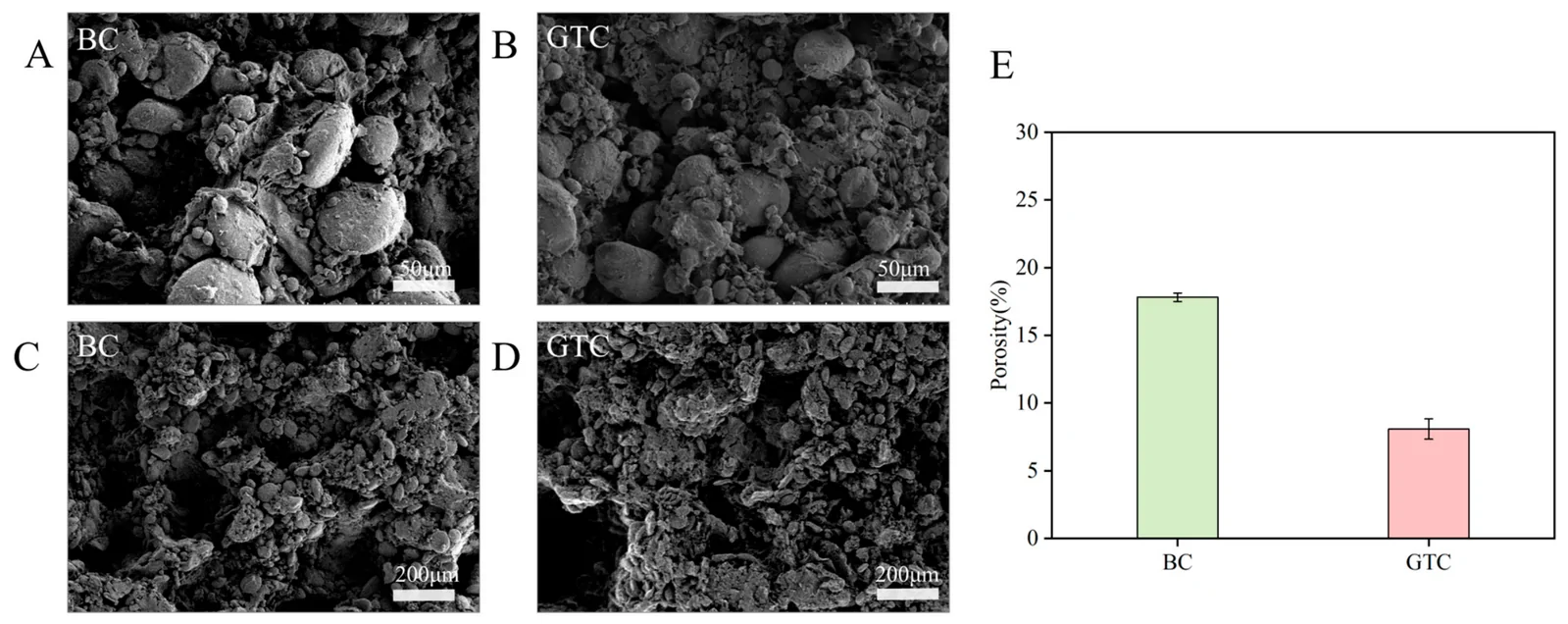
Scanning electron microscopy (SEM) images and porosity analysis of BC and GTC. (**A**,**C**) SEM images of BC at 250× and 1000× magnification, respectively; (**B**,**D**) SEM images of GTC at 250× and 1000× magnification, respectively; (**E**) Porosity determined by ImageJ image analysis. The porosity values are descriptive observations and were not statistically compared.

The fracture-surface microstructure of GTC and BC was examined by SEM to support the textural observations (Figure 5). At 1000× magnification, clear differences were observed in the pore structures of the two groups of cookies (Figure 5C,D). On the surface of the BC, relatively intact starch granules were observed. Their outlines were distinct, and they were scattered throughout the sample, suggesting that starch granule disruption during baking was limited. The microstructure exhibited distinct pores and a loose network, with a low degree of intergranular bonding. In the GTC (Figure 5D), the outlines of the starch granules became blurred, with a large amount of fine material adhering to their surfaces. Some granules were embedded in a continuous matrix, and the number of individual granules appeared markedly reduced, suggesting possible reorganization and enhanced integration of the internal cookie matrix following GTP addition. At 250× magnification, the BC exhibited a relatively loose network structure, with numerous pores and cavities in the interior, and relatively loose bonding between starch granules. In contrast, GTC exhibited a denser microstructure, with fewer pores and enhanced integration among starch granules and surrounding matrix components, resulting in a more continuous structure. The porosity of GTC (8.0%) was lower than that of BC (17.8%), a finding consistent with the higher hardness and breaking work of GTC in the texture analysis. These porosity values are descriptive observations from images of a single specimen per treatment.

The denser matrix and reduced pore volume observed in the SEM images could be one of the factors contributing to the higher resistance of the GTC structure to deformation and fracture, and thus to its higher hardness and breaking work, although a contribution of physical filler effects cannot be excluded. These findings provide microstructural observations consistent with the observed textural changes, while the potential molecular basis of the matrix restructuring was further investigated by FTIR analysis.

### 3.7. FTIR Analysis of Cookies

**Figure 6:**
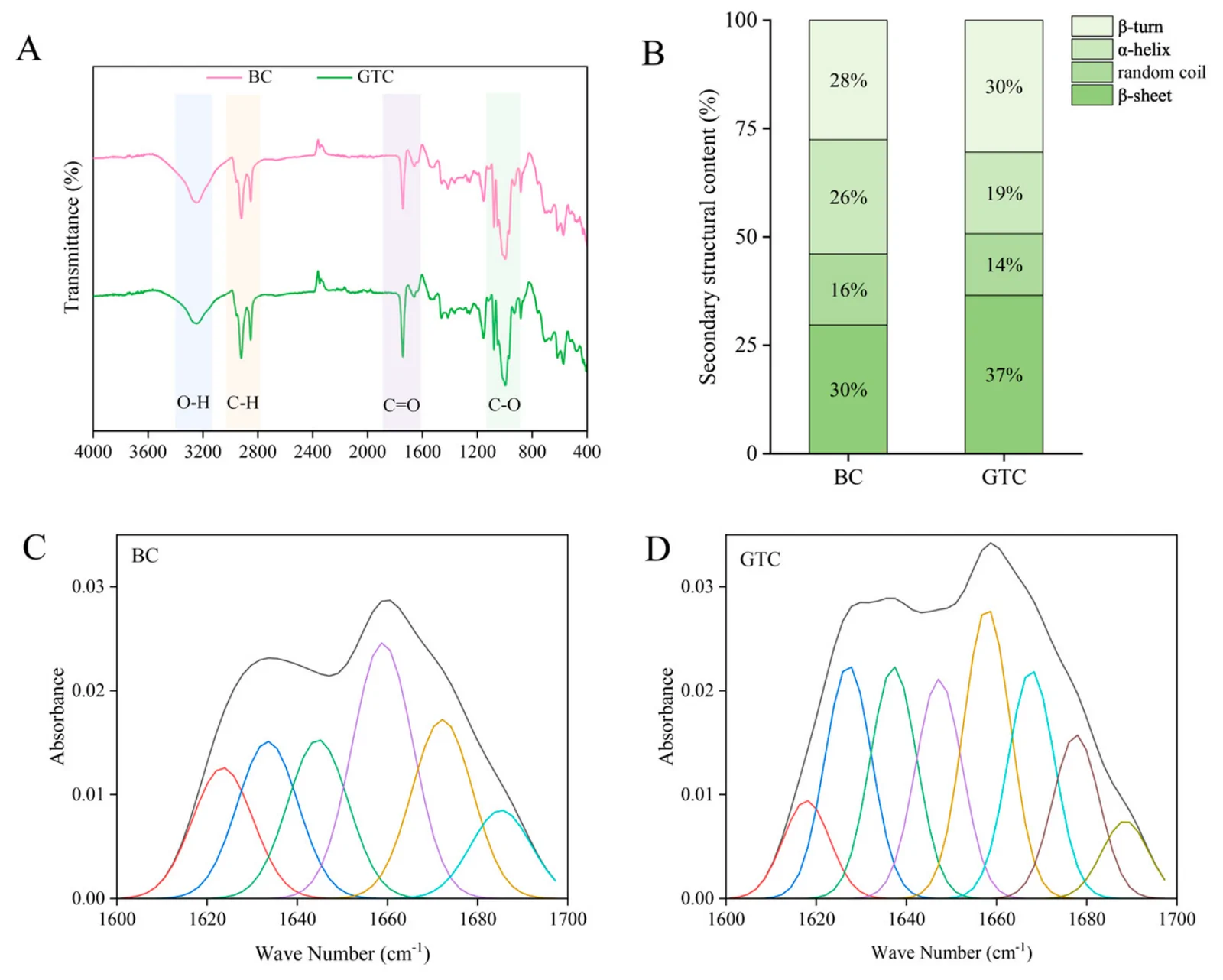
Fourier transform infrared (FTIR) spectra and protein secondary structure analysis of BC and GTC. (**A**) FTIR spectra of BC and GTC; (**B**) Relative contents of protein secondary structures obtained by peak fitting of the amide I region (1600–1700 cm^−1^); (**C**) Peak deconvolution of the amide I region for BC; (**D**) Peak deconvolution of the amide I region for GTC. The colored Gaussian curves represent β-sheet (1610–1640 cm^−1^), random coil (1640–1650 cm^−1^), α-helix (1650–1660 cm^−1^), and β-turn (1660–1670 cm^−1^). The solid black line represents the original spectrum.

To investigate the effect of GTP incorporation on the chemical structure and molecular interactions of the cookie matrix, FTIR spectroscopy combined with peak fitting of the amide I region was performed (Figure 6). The overall FTIR profiles of BC and GTC were highly similar, with no obvious new characteristic absorption bands observed after GTP incorporation. This indicates that GTP addition did not result in substantial changes in the major chemical functional groups of the cookie matrix, but may have modified the interactions among its existing components.

Both samples exhibited a broad and intense O–H stretching band near 3240 cm^−1^, mainly associated with hydroxyl groups in starch, proteins, and tea-derived phenolic compounds. Slight differences in the band profile were observed between BC and GTC, suggesting changes in the hydrogen-bonding environment after GTP incorporation. The C–H stretching band near 2920 cm^−1^ was chiefly linked to alkyl groups from lipids and carbohydrates. The band at about 1740 cm^−1^ was assigned to the C=O stretching of ester carbonyl groups, mostly originating from lipids. Additionally, the intense absorptions at 1000–1200 cm^−1^ were chiefly associated with C–O and C–O–C vibrations, indicating that GTP incorporation did not substantially modify the major functional groups of the starch–lipid matrix. These spectral features suggest that the effects of GTP were more likely associated with changes in intermolecular interactions rather than the formation of new covalent functional groups [48,49,50,51].

The relative contents of protein secondary structures were derived from deconvolution of the amide I band of one representative spectrum per treatment (*R*^2^ > 0.90). Given the complexity of the cookie matrix, these percentages should be regarded as exploratory estimates rather than definitive evidence of protein conformational changes. Peak fitting of the amide I region (1600–1700 cm^−1^) further suggested changes in the protein secondary structure following GTP incorporation (Figure 6B–D). Compared with BC, GTC showed lower proportions of α-helix (26% to 19%) and random coil (16% to 14%), whereas the proportions of β-sheet (30% to 37%) and β-turn (28% to 30%) increased. These changes suggest a possible redistribution of protein secondary structural elements following GTP incorporation, consistent with structural rearrangement of the protein conformation within the cookie matrix. Previous studies have shown that tea polyphenols associate with proteins chiefly via non-covalent forces, such as hydrogen bonds and hydrophobic interactions, potentially altering protein conformation and the organization of nearby matrix components [52].

The FTIR results were consistent with the SEM observations. GTC exhibited a denser and less porous microstructure than BC, while the changes in the amide I region suggested that GTP incorporation was accompanied by a redistribution of protein secondary structures. Although the amide I region may include minor contributions from other matrix components (e.g., starch and lipids), both treatments were analyzed under identical conditions, so the relative differences between BC and GTC remain interpretable. These observations are consistent with a possible restructuring of the matrix through changes in non-covalent interactions among tea polyphenols, proteins, and polysaccharide components, rather than through substantial changes in the major chemical functional groups of the system [51,52]. It should be noted, however, that SEM and FTIR demonstrate structural differences but do not directly establish this molecular mechanism. Such restructuring, if present, could be one of the factors contributing to the denser matrix observed by SEM and, consequently, to the increased hardness and breaking work of GTC.

Overall, the combined SEM and FTIR results provide complementary microstructural and molecular-level evidence for the structural changes induced by GTP incorporation. Nevertheless, given that the BC control was prepared by direct wheat-flour replacement and that FTIR band deconvolution was performed on the whole cookie powder, the present evidence cannot fully exclude the contribution of physical filler effects.

## 4. Conclusions

This study established an optimal formulation for erythritol-based green tea cookies (GTC) using single-factor experiments and response surface methodology (RSM). The optimized formulation consisted of 7.88 g GTP, 72.13 g butter, and 39.63 g erythritol, with a predicted sensory score of 80.35 and an actual score of 81.07 ± 1.87. Compared with the control butter cookies (BC), the optimized GTC exhibited modified nutritional composition, a darker green appearance, and distinct textural characteristics, including increased hardness, flexibility, and breaking work. In addition, GTP fortification markedly raised the TPC and improved the in vitro antioxidant capacity, as reflected in the significantly elevated DPPH and ABTS^+^ scavenging activities and the higher FRAP value. The stronger antioxidant performance of GTC in vitro was closely associated with its higher total phenolic content, underscoring the value of GTP as a natural antioxidant source for erythritol-based baked goods.

SEM analysis revealed that GTC exhibited a denser and more continuous cookie matrix than BC, with the porosity decreasing from 17.8% in BC to 8.0% in GTC. This microstructural change was consistent with the increased hardness and breaking work observed in GTC. FTIR analysis further suggested a possible redistribution of protein secondary structural elements, characterized by decreases in α-helix and random coil contents and increases in β-sheet and β-turn contents. Taken together, these observations suggest that the cookie matrix may have been restructured upon GTP incorporation, driven by altered non-covalent interactions between tea-derived compounds and the starch–protein matrix, rather than by major changes in chemical functional groups. It should be noted, however, that the SEM and FTIR data alone do not directly prove this molecular mechanism.

In addition, the present experimental design cannot fully distinguish molecular interactions from the physical filler effects of the replaced flour, and further molecular-level studies are warranted. Overall, GTP shows potential as a natural functional ingredient for developing erythritol-based cookies with desirable sensory properties and enhanced antioxidant capacity.

This study provides a sensory-driven formulation strategy and structural insights for the development of erythritol-based products incorporating GTP. Further research is needed to evaluate the storage stability, flavor characteristics, and digestive behavior of GTP-enriched erythritol-based cookies. In addition, the physicochemical measurements were performed on cookies from a single production batch. Therefore, the observed statistically significant differences reflect within-batch formulation differences, and studies using multiple independently prepared batches would be required to confirm the robustness of these findings across production runs.

## Figures and Tables

**Table 1:** Factors and levels of the Box–Behnken response surface design.

Level	Factor		
	GTP/g	Erythritol/g	Butter/g
−1	5.0	30.0	65.0
0	7.5	40.0	75.0
1	10.0	50.0	85.0

**Table 2:** Design and results of the response surface experiment.

Test No.	GTP/g	Erythritol/g	Butter/g	Sensory Score/Points
1	5.0	30.0	75.0	64.42
2	10.0	30.0	75.0	68.48
3	5.0	50.0	75.0	62.44
4	10.0	50.0	75.0	67.52
5	5.0	40.0	65.0	69.10
6	10.0	40.0	65.0	73.63
7	5.0	40.0	85.0	65.69
8	10.0	40.0	85.0	67.94
9	7.5	30.0	65.0	70.60
10	7.5	50.0	65.0	71.65
11	7.5	30.0	85.0	70.56
12	7.5	50.0	85.0	62.85
13	7.5	40.0	75.0	81.00
14	7.5	40.0	75.0	80.79
15	7.5	40.0	75.0	78.19
16	7.5	40.0	75.0	81.85
17	7.5	40.0	75.0	78.77

**Table 3:** Analysis of variance of the regression model.

Source	Sum of Squares	Degrees of Freedom	Mean Square	*F*-Value	*p*-Value	Significance
Regression Model	659.63	9	73.29	41.94	<0.0001	***
A	31.65	1	31.65	18.11	0.0038	**
B	11.54	1	11.54	6.60	0.0370	*
C	40.22	1	40.22	23.01	0.0020	**
AB	0.26	1	0.26	0.15	0.7120	
AC	1.29	1	1.29	0.74	0.4189	
BC	19.14	1	19.14	10.95	0.0130	*
A^2^	213.34	1	213.34	122.07	<0.0001	***
B^2^	223.77	1	223.77	128.03	<0.0001	***
C^2^	64.51	1	64.51	36.91	0.0005	***
Residuals	12.24	7	1.75			
Lack of Fit	2.45	3	0.82	0.33	0.8033	
Pure Error	9.79	4	2.45			
Sum	671.86	16				

Notes: df, degrees of freedom. Pure Error df = 4, derived from the five center-point replicates (5 − 1 = 4); Lack of Fit df = 3, calculated as the residual df (7) minus the Pure Error df (4). *, **, and *** indicate significance at *p* < 0.05, *p* < 0.01, and *p* < 0.001, respectively.

**Table 4:** Contents of basic nutritional components in cookies.

Component	BC	GTC
Moisture (%)	2.01 ± 0.12	2.41 ± 0.08 *
Total ash (%)	0.81 ± 0.02	1.02 ± 0.06 *
Crude fat (%)	19.19 ± 0.20	19.48 ± 0.40
Crude protein (%)	7.18 ± 0.01	7.38 ± 0.05
Total carbohydrate (%)	70.81 ± 0.29	69.72 ± 0.40 *
Energy (kcal/100 g)	418.67 ± 0.73	417.70 ± 2.16

Note: “*” indicates a significant difference between BC and GTC (*p* < 0.05).

**Table 5:** Color difference and texture of cookies.

Cookies	L*	a*	b*	Hardness (g)	Flexibility (mm)	Breaking Work (g·s)
BC	79.88 ± 0.53	1.84 ± 0.03	26.72 ± 0.91	1228.53 ± 21.47	0.23 ± 0.01	39.40 ± 2.72
GTC	53.29 ± 0.60 **	−2.34 ± 0.04 **	35.71 ± 0.23 **	1669.54 ± 13.43 **	0.29 ± 0.01 **	62.82 ± 4.20 **

Note: “**” indicates a highly significant difference (*p* < 0.01).

## Data Availability

The original contributions presented in the study are included in the article/Supplementary Materials; further inquiries can be directed to the corresponding authors.

## Supplementary Materials

The following supporting information can be downloaded at: https://www.mdpi.com/article/10.3390/foods15213776/s1, Table S1: Sensory Evaluation Criteria for Green Tea Cookies; Table S2: Sensory Evaluation Scoring Sheet for Green Tea Cookies; Table S3: Summary of model diagnostics for the sensory score response; Figure S1: Residual diagnostic plots for the sensory score model. (A) Normal probability plot of externally studentized residuals; (B) Externally studentized residuals versus predicted values.

## Abbreviations

The following abbreviations are used in this manuscript:

ABTS	2,2′-azino-bis(3-ethylbenzothiazoline-6-sulfonic acid)
BC	Butter cookie
DPPH	2,2-diphenyl-1-picrylhydrazyl
FRAP	Ferric Reducing Antioxidant Power
FTIR	Fourier transform infrared
GTC	Green tea cookie
GTP	Green tea powder
RSM	Response surface methodology
SEM	Scanning Electron Microscopy
TPC	Total phenolic content

## References

[1] Koh W.Y., Lim X.X., Tan T.C., Mamat H., Kobun R., Rasti B. (2023). Utilising Spent Tea Leaves Powder as Functional Ingredient to Enhance the Quality of Non-Gluten Shortbread Cookies. Foods.

[2] Vishwakarma K., Chandra Sekaran V., Patwardhan V., Kamath A. (2024). A bibliometric review of functional ingredients and their efficacy in developing functional biscuits. F1000Research.

[3] Huang X., Li A., Zhou X., Li P., Huang J., Shao L., Pei Y. (2026). Formulation Optimization, Quality Characterization, and Flavor Profiling of Cookies Enriched with Ultrafine Dark Tea Powder. Foods.

[4] Tolve R., Simonato B. (2024). Impact of Functional Ingredients on the Technological, Sensory, and Health Properties of Bakery Products. Foods.

[5] Phongnarisorn B., Orfila C., Holmes M., Marshall L.J. (2018). Enrichment of Biscuits with Matcha Green Tea Powder: Its Impact on Consumer Acceptability and Acute Metabolic Response. Foods.

[6] Salihu S., Gashi N., Hasani E. (2023). Effect of plant extracts addition on the physico-chemical and sensory properties of biscuits. Appl. Sci..

[7] Zarzycki P., Wirkijowska A., Pankiewicz U. (2024). Functional bakery products: Technological, chemical and nutritional modification. Appl. Sci..

[8] Dhami K., Thapa P., Kumar S., Semwal P., Rai N., Anand J. (2025). Green tea-millets based multi-nutritive cookies: Innovative formulation and nutritional analyses. Food Chem. X.

[9] Zhang G., Zhong Y., Zhang X., Wang Y., Sun Y., Li X., Liu Z., Liang J. (2024). Flavor Characteristics, Antioxidant Activity and In Vitro Digestion Properties of Bread with Large-Leaf Yellow Tea Powder. Foods.

[10] Gramza-Michałowska A., Kobus-Cisowska J., Kmiecik D., Korczak J., Helak B., Dziedzic K., Górecka D. (2016). Antioxidative potential, nutritional value and sensory profiles of confectionery fortified with green and yellow tea leaves (*Camellia sinensis*). Food Chem..

[11] Tyagi P., Chauhan A.K. (2020). Optimization and characterization of functional cookies with addition of *Tinospora cordifolia* as a source of bioactive phenolic antioxidants. LWT.

[12] Hong J., An D., Wang M., Liu C., Buckow R., Li L., Zheng X., Bian K. (2021). Wheat noodles enriched with A-type and/or B-type wheat starch: Physical, thermal and textural properties of dough sheet and noodle samples from different noodle-making process. Int. J. Food Sci. Technol..

[13] Ahmad M., Baba W.N., Wani T.A., Gani A., Gani A., Shah U., Wani S.M., Masoodi F.A. (2015). Effect of green tea powder on thermal, rheological & functional properties of wheat flour and physical, nutraceutical & sensory analysis of cookies. J. Food Sci. Technol..

[14] Susanti S., Nurwantoro D.H.W., Suryani A.E., Nisa K., Katherinatama A., Asikin Y. (2025). Antioxidant properties, functional components, and sensory profile of green tea oatmeal cookies. Prog. Nutr..

[15] Dana H., Sonia A. (2024). Substituting sugar in pastry and bakery products with functional ingredients. Appl. Sci..

[16] Mazi T.A., Stanhope K.L. (2023). Erythritol: An In-Depth Discussion of Its Potential to Be a Beneficial Dietary Component. Nutrients.

[17] De Vivo A., Balivo A., Esposito F., Romano R., Cirillo T., Sarghini F., Genovese A. (2026). Mitigation of acrylamide in cookies through partial replacement of conventional sugars with erythritol and maltitol. Food Chem..

[18] Różańska M.B., Rychlewski P., Mildner-Szkudlarz S., Muzolf-Panek M. (2025). Formation rates of selected Maillard reaction products in model gluten-free bread, with consideration of erythritol: A preliminary study. LWT.

[19] Struck S., Jaros D., Brennan C.S., Rohm H. (2014). Sugar replacement in sweetened bakery goods. Int. J. Food Sci. Technol..

[20] Blasi E., Rossi E.S., Pietrangeli R., Nasso M., Cicatiello C., Palombieri S., Sestili F. (2024). Functional Biscuits, a Healthy Addition to Your Coffee Break-Evaluating Consumer Acceptability and Willingness to Pay. Foods.

[21] Guiné R.P., Florença S.G. (2024). Development and characterisation of functional bakery products. Physchem.

[22] Raczkowska E., Wojdyło A., Nowicka P. (2024). The use of blackcurrant pomace and erythritol to optimise the functional properties of shortbread cookies. Sci. Rep..

[23] Cui H., Zhang J., Yu J., Jiang H., Ao C., Huang H. (2019). Processing technology of tea bakery foods—A Review. Czech J. Food Sci..

[24] Seog E.J., Kim H.R., Lee J.H. (2008). Optimization of onion cookie baking process using response surface methodology. J. Food Qual..

[25] (2016). National Food Safety Standard: Determination of Moisture in Food.

[26] (2025). National Food Safety Standard: Determination of Protein in Food.

[27] (2025). National Food Safety Standard: Determination of Fat in Food.

[28] (2016). National Food Safety Standard: Determination of Ash in Food.

[29] (2018). Determination of Total Polyphenols and Catechins Content in Tea.

[30] Brand-Williams W., Cuvelier M.-E., Berset C. (1995). Use of a free radical method to evaluate antioxidant activity. LWT.

[31] Re R., Pellegrini N., Proteggente A., Pannala A., Yang M., Rice-Evans C. (1999). Antioxidant activity applying an improved ABTS radical cation decolorization assay. Free Radic. Biol. Med..

[32] Benzie I.F., Strain J.J. (1996). The ferric reducing ability of plasma (FRAP) as a measure of “antioxidant power”: The FRAP assay. Anal. Biochem..

[33] Honrado A., Ardila P., Lecinena P., Beltrán J.A., Calanche J. (2024). Nutritional enrichment of cereal foods: Feasibility study and characterisation of biscuits fortified with seabass by-products. Int. J. Food Sci. Technol..

[34] Otsu N. (1979). A threshold selection method from gray-level histograms. IEEE Trans. Syst. Man Cybern..

[35] Saberi F., Kouhsari F., Gasparre N. (2024). Green alternative for sodium metabisulfite substitution: Comparison of bacterial and fungal proteases effect in hard biscuit making. Food Sci. Technol. Int..

[36] Sharifi M., Moshfegh N., Abbasi A. (2025). Studying Several Properties of Emulgel Based on κ-Carrageenan and Its Incorporation as Shortening Material in Cookies. Food Sci. Nutr..

[37] Jiang G., Wu Z., Ameer K., Song C. (2021). Physicochemical, antioxidant, microstructural, and sensory characteristics of biscuits as affected by addition of onion residue. J. Food Meas. Charact..

[38] Susanti S., Bintoro V.P., Katherinatama A., Arifan F. (2021). Chemical, physical and hedonic characteristics of green tea powder fortified oatmeal cookies. Food Res..

[39] Lei Y., Guo X., Wu Z., Feng J., Yang T., Zhang W., Cong Y. (2025). Evaluation of flavor quality of commercial butter: A systematic comparison of sweet cream and cultured butter. Food Chem. X.

[40] Laguna L., Varela P., Salvador A., Fiszman S. (2013). A new sensory tool to analyse the oral trajectory of biscuits with different fat and fibre contents. Food Res. Int..

[41] Arepally D., Reddy R.S., Goswami T.K., Datta A.K. (2020). Biscuit baking: A review. LWT.

[42] Hu H., Wang Y., Shen M., Huang Y., Li C., Nie S., Xie M. (2022). Effects of baking factors and recipes on the quality of butter cookies and the formation of advanced glycation end products (AGEs) and 5-hydroxymethylfurfural (HMF). Curr. Res. Food. Sci..

[43] Lin S. (2022). Dietary fiber in bakery products: Source, processing, and function. Adv. Food. Nutr. Res..

[44] Ramos P., Oliveira T., Fernandes A., Souza L., Castro J. (2024). Nutritional quality of bakery products enriched with alternative flours. Int. J. Fam. Community Med..

[45] Olivier J., Symington E.A., Jonker C.Z., Van Eeden T.S., Rampedi I.T. (2012). Comparison of the mineral composition of leaves and infusions of traditional and herbal teas. S. Afr. J. Sci..

[46] Li J., Ma J., Li Q., Fan S., Fan L., Ma H., Zhang Y., Zheng L. (2021). Determination of 35 Free Amino Acids in Tea Using Ultra-Performance Liquid Chromatography Coupled with Quadrupole Time-of-Flight Mass Spectrometry. Front. Nutr..

[47] Sharma A., Zhou W. (2011). A stability study of green tea catechins during the biscuit making process. Food Chem..

[48] Yan Z., Zhong Y., Duan Y., Chen Q., Li F. (2020). Antioxidant mechanism of tea polyphenols and its impact on health benefits. Anim. Nutr..

[49] Wang W., Le T., Wang W., Yu L., Yang L., Jiang H. (2023). Effects of Key Components on the Antioxidant Activity of Black Tea. Foods.

[50] Wang Z., Han Z., Zhang L. (2025). Advances and Outlook of Tea Polyphenol Chemistry: A Review. J. Agric. Food Chem..

[51] Bandyopadhyay P., Ghosh A.K., Ghosh C. (2012). Recent developments on polyphenol–protein interactions: Effects on tea and coffee taste, antioxidant properties and the digestive system. Food Funct..

[52] Niu J., Shang M., Li X., Sang S., Chen L., Long J., Jiao A., Ji H., Jin Z., Qiu C. (2024). Health benefits, mechanisms of interaction with food components, and delivery of tea polyphenols: A review. Crit. Rev. Food Sci. Nutr..

