# Supplementary material for: Sensory-Driven Formulation Optimization, Physicochemical Properties, Antioxidant Activity, and Structural Characterization of Erythritol-Based Green Tea Cookies
Source: Foods. 2026 Oct 22;15(21):3776. doi: 10.3390/foods15213776 (PMC13763050; doi:10.3390/foods15213776)
Supplement: Supplementary file 1 [file foods-15-03776-s001.zip › foods-4597036-supplementary.pdf]

Supplementary Materials

Table S1 Sensory Evaluation Criteria for Green Tea Cookies

Attribute	Evaluation criteria	Score range
Shape and Appearance	Complete appearance, uniform thickness, and no obvious defects	17–20
	Relatively complete appearance with slight differences in thickness	13–16
	Obvious damage and non-uniform shape	9–12
	Severe damage and extremely non-uniform thickness	5–8
	Basic shape unrecognizable	1–4
Color	Natural yellowish-green color, uniform, with slight gloss	17–20
	Slightly dark yellowish-green, acceptable color, slightly lacking gloss	13–16
	Dark or too pale color with uneven distribution	9–12
	Unnatural color without gloss	5–8
	Abnormal color that affects sensory perception	1–4
Aroma	Rich and pure tea aroma without off-flavor	17–20
	Distinct tea aroma without off-flavor	13–16
	Slight and somewhat weak tea aroma	9–12
	Impure aroma or slight foreign odor	5–8
	Off-flavor or severe lack of tea aroma	1–4
Taste	Crisp and pleasant, moderate sweetness and tea flavor, free of stickiness and grittiness	17–20
	Slightly sticky or slightly unbalanced sweetness, with slight bitterness	13–16
	Too hard or too soft, unbalanced sweetness and bitterness, with grittiness	9–12
	Markedly sticky or rough mouthfeel	5–8
	Clearly unpleasant to eat, e.g., raw dough or hard lumps	1–4
Texture	Fine and layered cross-section without large cracks or cavities	17–20
	Slightly rough cross-section with acceptable structure	13–16
	Insufficient layering with small cracks or pores	9–12
	Obvious cracks or cavities with loose structure	5–8
	Loose or severely collapsed structure	1–4

Table S2 Sensory Evaluation Scoring Sheet for Green Tea Cookies

Butter/ Erythritol/ Green Tea Powder/ Texture	Shape and appearance (20%)	Color (15%)	Aroma (15%)	Taste (30%)	Texture (20%)

Note: Panelists scored each attribute of the samples on a 1–20 scale according to the evaluation criteria given in Table S1.

Table S3 Summary of model diagnostics for the sensory score response

Statistic	Value
R^2	0.98
Adjusted R^2	0.96
Predicted R^2	0.92
Std. Dev.	1.32
Mean	71.50
Coefficient of variation (C.V. %)	1.85
Adequate precision	17.61
Lack-of-fit (p-value)	0.80

Note: CV (%) = (Std. Dev./Mean) × 100; an adequate precision value greater than 4 indicates an adequate signal-to-noise ratio.

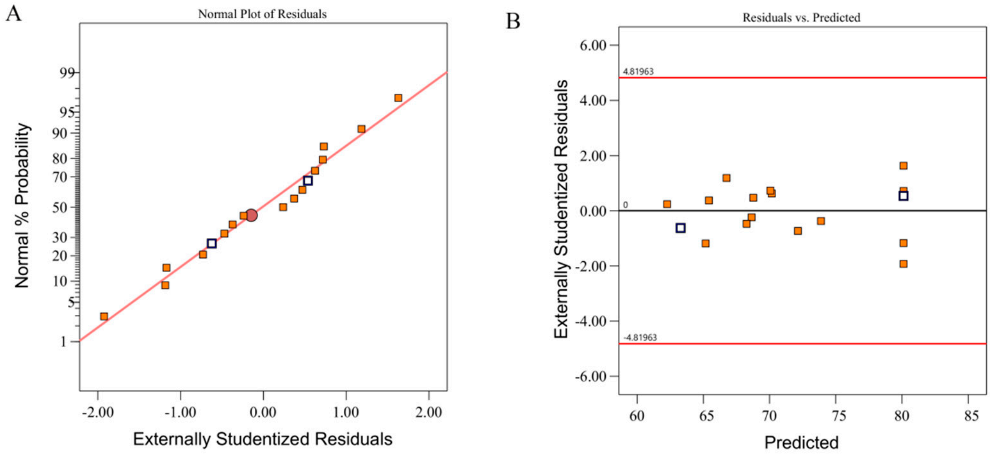

Figure S1. Residual diagnostic plots for the sensory score model. (A) Normal probability plot of externally studentized residuals; (B) Externally studentized residuals versus predicted values.
